# Supplementary material for: COZOID: contact zone identifier for visual analysis of protein-protein interactions
Source: BMC Bioinformatics. 2018 Apr 6;19:125. doi: 10.1186/s12859-018-2113-6 (PMC5889581; doi:10.1186/s12859-018-2113-6)
Supplement: Supplementary file 2 — Software build. Executable binary file of the software tool. (ZIP 118,784 kb) [file 12859_2018_2113_MOESM2_ESM.zip › cozoid/java/docs/beansbinding-1.2.1-doc.zip!/org/jdesktop/swingbinding/impl/class-use/AbstractColumnBinding.html]

Uses of Class org.jdesktop.swingbinding.impl.AbstractColumnBinding (Beans Binding)


---


|  |  |  |
| --- | --- | --- |
| **Overview** \| **Package** \| **Class** \| **Use** \| **Tree** \| **Deprecated** \| **Index** \| **Help** | |  |
| PREV   NEXT | **FRAMES**    **NO FRAMES**     **All Classes** |  |


---


## **Uses of Class org.jdesktop.swingbinding.impl.AbstractColumnBinding**

| Packages that use AbstractColumnBinding | |
| --- | --- |
| **org.jdesktop.swingbinding** | Provides support for binding to complex Swing components, and documentation on the interesting Swing properties to bind to. |
| **org.jdesktop.swingbinding.impl** | Private implementation details for Swing binding. |

Uses of AbstractColumnBinding in org.jdesktop.swingbinding

| Subclasses of AbstractColumnBinding in org.jdesktop.swingbinding | |
| --- | --- |
| `class` | `JListBinding.DetailBinding`             `DetailBinding` represents a binding between a property of the elements in the `JListBinding's` source `List`, and the values shown in the `JList`. |
| `class` | `JTableBinding.ColumnBinding`             `ColumnBinding` represents a binding between a property of the elements in the `JTableBinding's` source `List`, and a column in the table. |

Uses of AbstractColumnBinding in org.jdesktop.swingbinding.impl

| Methods in org.jdesktop.swingbinding.impl that return AbstractColumnBinding | |
| --- | --- |
| `protected abstract  AbstractColumnBinding[]` | `ListBindingManager.getColBindings()` |

---


|  |  |  |
| --- | --- | --- |
| **Overview** \| **Package** \| **Class** \| **Use** \| **Tree** \| **Deprecated** \| **Index** \| **Help** | |  |
| PREV   NEXT | **FRAMES**    **NO FRAMES**     **All Classes** |  |


---
